# Supplementary material for: Proteomic Signatures of the Serological Response to Influenza Vaccination in a Large Human Cohort Study
Source: Viruses. 2022 Nov 9;14(11):2479. doi: 10.3390/v14112479 (PMC9696600; doi:10.3390/v14112479)
Supplement: Supplementary file 1 [file viruses-14-02479-s001.zip › viruses-1943578-supplementary.pdf]

Supplementary Material

Shaohuan Wu[1#*], Smruti Pushalkar[1#], Shuvadeep Maity[1,4], Matthew Pressler[1], Justin Rendleman[1], Burcu Vitrinel[1], Michael Carlock[2,3], Ted Ross[2,3], Hyungwon Choi[5], and Christine Vogel[1*]

1 Center for Genomics and Systems Biology, New York University, NY, USA

2 Department of Infectious Diseases, College of Veterinary Medicine, University of Georgia, Athens, GA, USA

3 Center for Vaccines and Immunology, University of Georgia, Athens, GA, USA

4 Birla Institute of Technology and Science (BITS)-Pilani (Hyderabad Campus), Hyderabad, India

5 Department of Medicine, Yong Loo Lin School of Medicine, National University of Singapore, Singapore City, Singapore

# Equally contributing authors

* Corresponding author. E-mail: sw5019@nyu.edu, cvoge@nyu.edu

Supplementary Data File S1

This file contains information on demographic and other factors recorded for the flu vaccine recipients, protein level data, and statistical testing results.

Supplementary Figure S1. Demographic and other factors across 138 participants ranked by their adjusted seroconversion values

The data are equivalent to what is shown in **Figure 1**, but sorted by adjusted seroconversion to illustrate its independence from the other factors. Note that the remaining correlation with age is due to additional (non-linear) effects that the correction did not capture [1]. Adj. seroconversion, adjusted seroconversion. Feb, February. Sep, September. Vacc, vaccination.

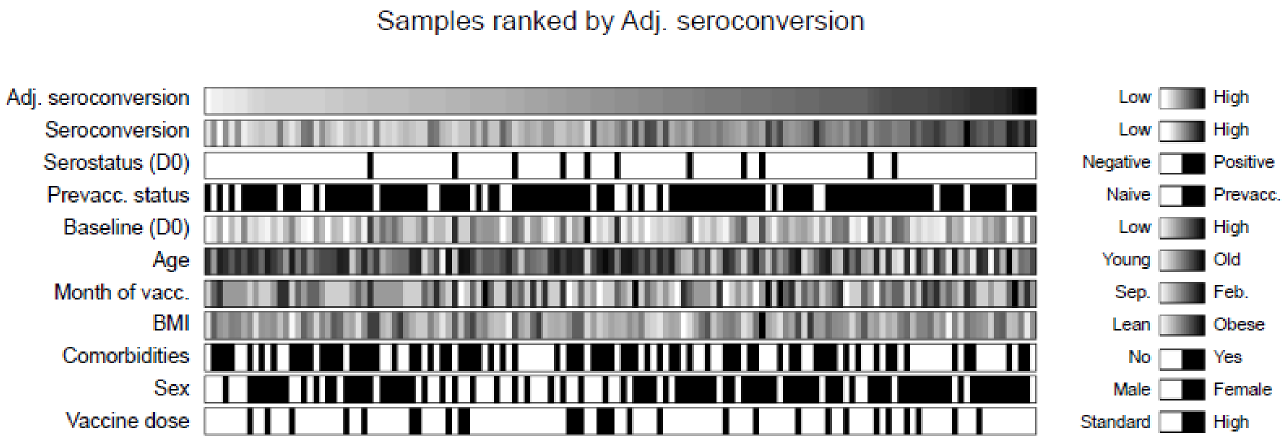

Supplementary Figure S2. protein levels levels for LRG and FHR4 in participants grouped by body mass index

The box and whisker plots show the distributions of the respective normalized protein levels values for LRG (**A**) and FHR4 (**B**) for two groups of participants: lean and obese with a body mass index of <25 and >30, respectively. P-values are calculated from a t-test. As both proteins link to obesity, the observed difference in protein levels confirms the quality of the data.

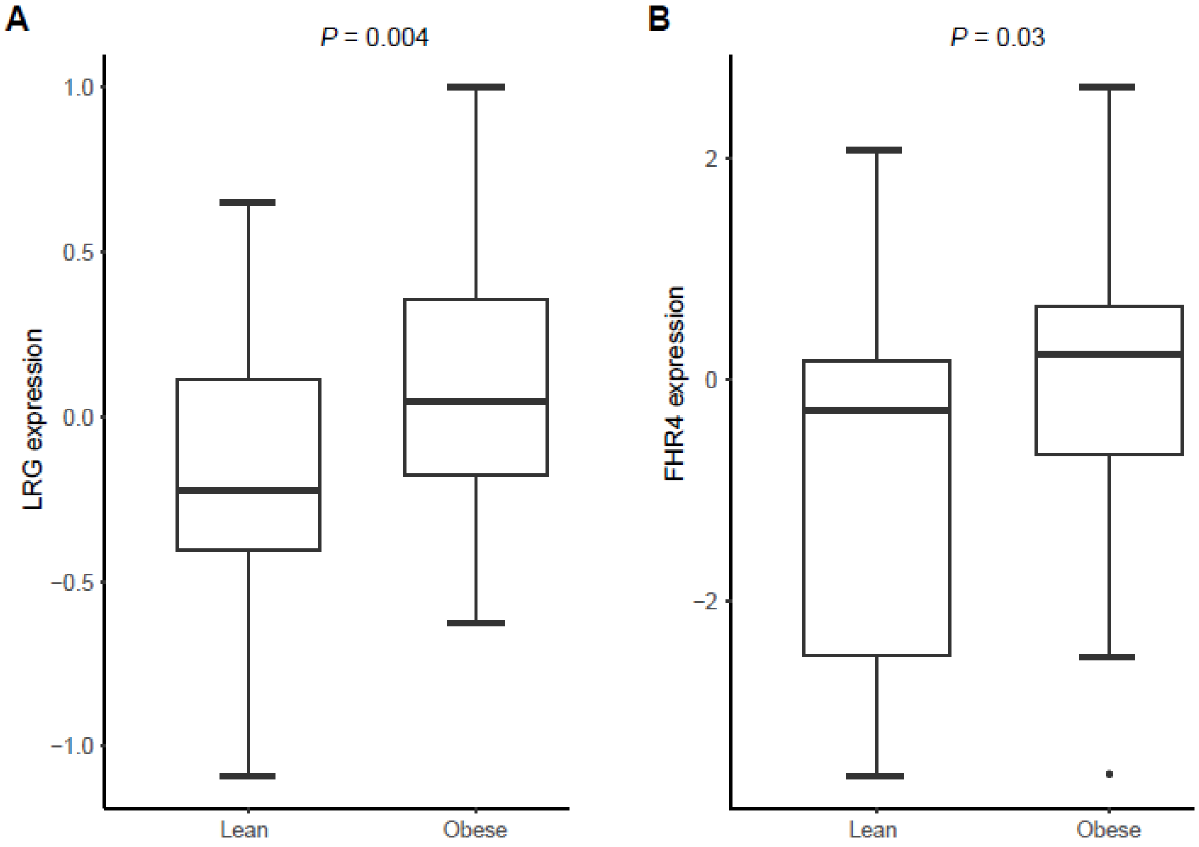

Supplementary Figure S3. The correlation-based method confirms results from testing extreme values

The figures show the distributions of Spearman's correlation values between the respective protein's level and raw seroconversion (**A**) and adjusted seroconversion (**B**). Genes in cholesterol metabolism (**A**) and regulation of actin cytoskeleton (**B**) pathways are labeled red. Adj. seroconversion, adjusted seroconversion. Corr. coef, correlation coefficient. Expr, expression.

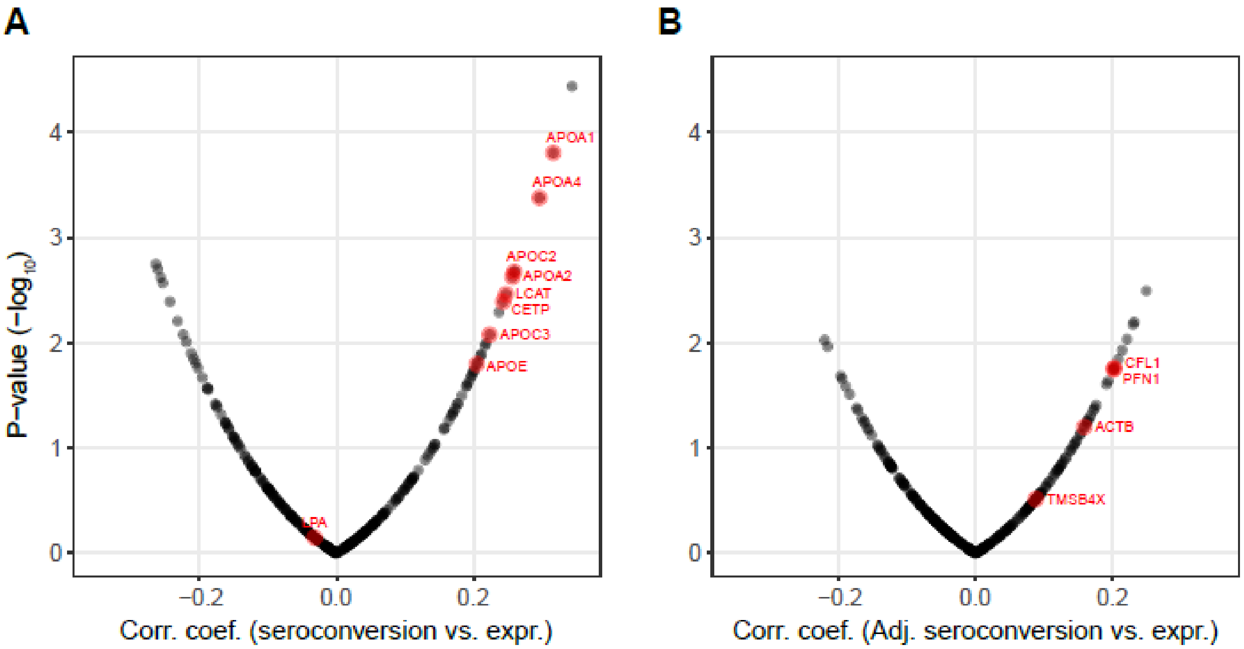

Supplementary Figure S4. Levels of proteins in participants with high and low adjusted seroconversion, plotted for each of the four vaccine strains

A-D. Protein level fold change between top and bottom 30 seroconverters (adjusted seroconversion) for four strains. Red labels mark the four genes from the regulation of actin cytoskeleton pathway that was a significantly enriched pathway in the gene set enrichment analysis of the overall comparison (all strains combined, false discovery rate < 0.05). Only for the H3N2 strain, the four genes show substantial differences in protein levels between high and low seroconverters. Adj. seroconversion, adjusted seroconversion.

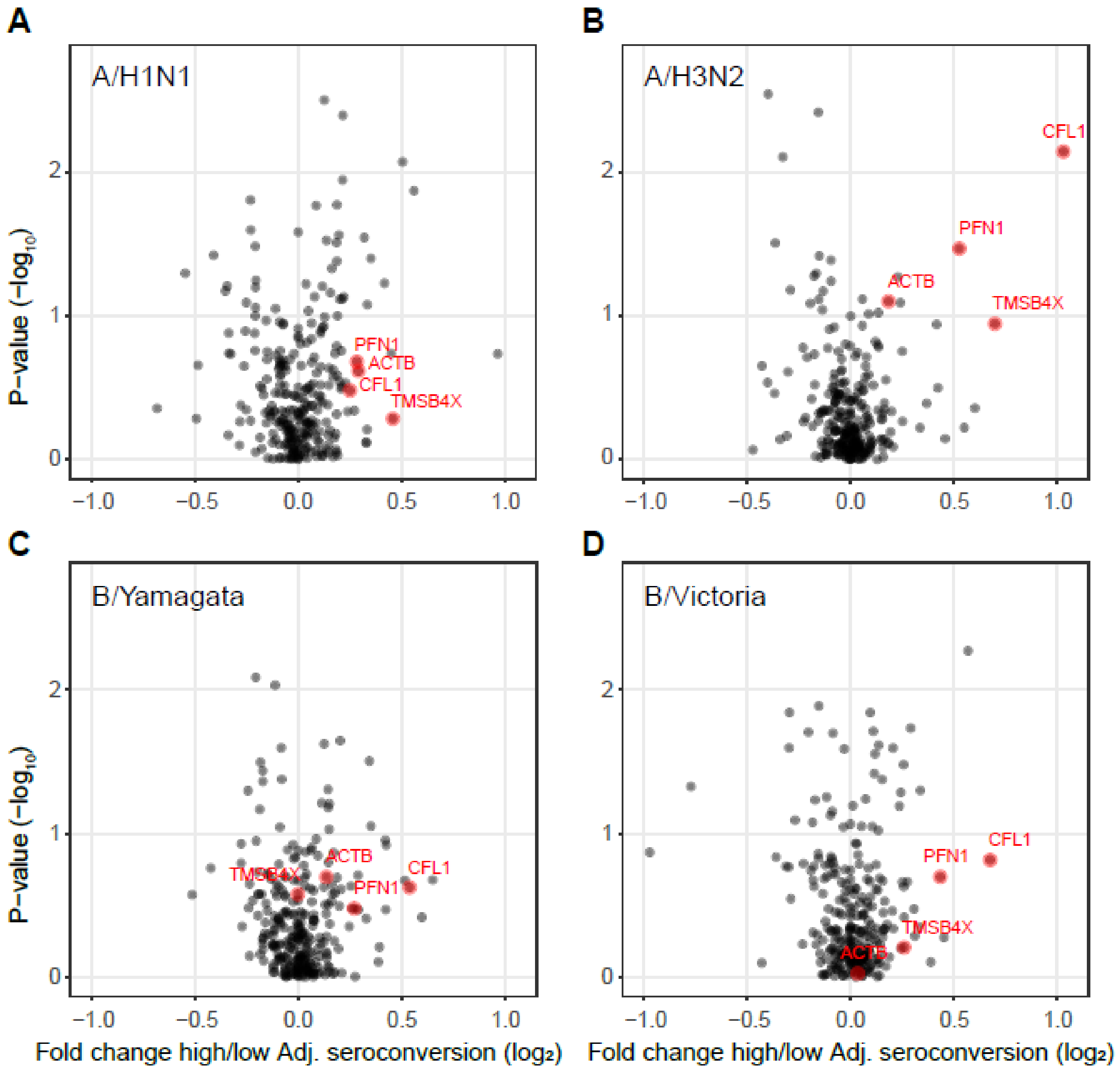

Supplementary Figure S5. PCA result based on the protein level data.

The first 100 principal components are shown across the participants. The first principal component (PC1, first row in the heatmap shown in the lower panel) is not associated with any of the variables shown in the upper panel.

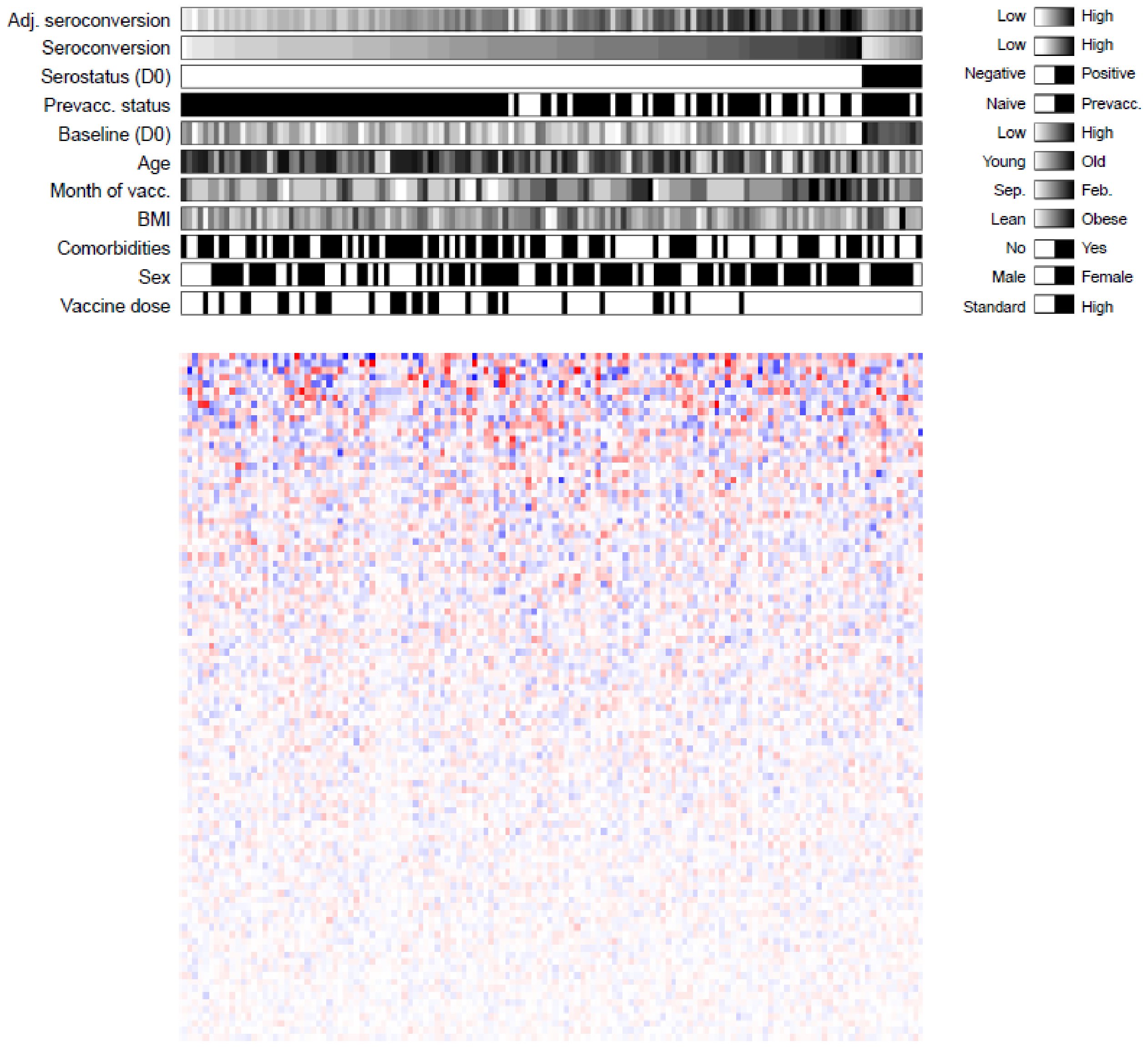

References

1. Wu, S.; Ross, T.M.; Carlock, M.A.; Ghedin, E.; Choi, H.; Vogel, C. Evaluation of Determinants of the Serological Response to the Quadrivalent Split-inactivated Influenza Vaccine. *Molecular Systems Biology* 2022, *18*.
